# The burden of pancreatic cancer in Latin America and the Caribbean: trends in incidence, mortality and DALYs from 1990 to 2019

**DOI:** 10.3332/ecancer.2025.1827

**Published:** 2025-01-16

**Authors:** Diego Rodrigues Mendonça e Silva, Max Moura de Oliveira, Gisele Aparecida Fernandes, Maria Paula Curado

**Affiliations:** 1 Postgraduate Program in Epidemiology, School of Public Health, University of São Paulo, São Paulo, SP 01246-904, Brazil; 2 Hospital Cancer Registry, A.C.Camargo Cancer Center, São Paulo, SP 01246-904, Brazil; 3 Department of Collective Health, Institute of Tropical Pathology and Public Health, Federal University of Goias, Goiania, GO 01246-904, Brazil; 4 Group of Epidemiology and Statistics on Cancer, A.C.Camargo Cancer Center, São Paulo, SP 01246-904, Brazil; a https://orcid.org/0000-0001-8469-8415; b https://orcid.org/0000-0002-0804-5145; c https://orcid.org/0000-0002-5978-3279; d https://orcid.org/0000-0001-8172-2483

**Keywords:** public health, epidemiology, pancreatic cancer, global burden disease

## Abstract

This study analyzed the burden of pancreatic cancer by investigating its incidence, mortality and disability-adjusted life years (DALYs), as well as the proportion of pancreatic cancer deaths attributable to behavioural and metabolic risk factors in Latin America and the Caribbean (LAC) countries.

**Methods:**

This study focuses on pancreatic cancer using the Global Burden of Disease 2019 study database. Results were described for 23 LAC countries for 1990–2019, evaluating their age-standardised incidence rates, mortality rates, DALYs, average annual percent change and the fraction of deaths attributable to behavioural and metabolic risk factors.

**Results:**

We observed that in LAC, pancreatic cancer incidence rates ranged from 1.2 in Haiti to 15.8/100,000 in Uruguay among men. The highest increase in incidence rate was observed in Trinidad and Tobago: 7.7% per year. The mortality rate was higher in Uruguay and lower in Haiti, for both sexes. The highest rise in the numbers of DALYs in 2019 was observed in Brazil and Mexico. The proportion of pancreatic cancer deaths attributable to smoking was reduced between 1990 and 2019 for both sexes in LAC countries; however, it increased for metabolic risk factors.

**Conclusion:**

The increasing trend in pancreatic cancer observed in LAC may be associated with a rise in risk factors such as high fasting plasma glucose and high body mass index in both sexes. This trend will likely have a substantial impact on the healthcare system in the coming decades.

## Introduction

In 2020, pancreatic cancer accounted for almost as many deaths (466,000) as cases (496,000) worldwide. This type of cancer is the seventh leading cause of cancer death among both sexes. The highest incidence rates in 2020 from Globocan, were in Eastern Europe, with 9.9/100,000, followed by Western Europe (9.8) and Northern America (9.3). Average incidence was observed in South America (5.4) and in Central America (3.8). The lowest incidence was reported in Middle and Eastern Africa (2.0), as well as in South-Central Asia (1.5) among men [1]. The GDB 2019 results described the incidence of 11.5/100,000 in both sexes and 11.9/100,000 for mortality in Argentina, followed by 7.5 for incidence and 7.8 for mortality in Chile, 6.2/100,000 and 6.5/100,000 respectively in Brazil, within Mexico was 5.8 for incidence and 6.0 for mortality [2]. In Latin America and the Caribbean (LAC), projections from 2018 to 2040 estimate a 99.3% increase in incidence and a 101.0% increase in mortality [3].

In high/very high Human Development Index (HDI) countries, the age-standardised incidence rate (ASIR) for pancreas cancer is 7.2 and the mortality rate is 6.7, whereas in low/medium HDI regions, the incidence is 1.6 and the mortality rate is 1.5/100,000 among men. Among women, the incidence is 5.0, and the mortality rate is 4.6/100,000 in high/very high HDI regions and 1.0/100,000 in low/medium HDI regions. Rates are 4-fold to 5-fold higher in higher HDI countries, with the highest incidence rates in Europe, Northern America and Australia/New Zealand [1, 4].

It is estimated that the incidence and mortality of pancreatic cancer will continue to increase for the next 20 years. This will cause a huge economic burden to populations worldwide [5–7]. Recent studies on the burden of pancreatic cancer in LAC have been scarce. It is possible to better understand the impact of this malignancy in these populations using the Global Burden of Disease (GBD) study database [8]. This study analyzed the burden of pancreatic cancer in LAC countries for incidence, mortality, disability-adjusted life years (DALYs) and the fraction of pancreatic cancer age-standardised deaths attributable to behavioral and metabolic risk factors, through the GBD database between 1990 and 2019.

## Methods

This study was based on the GBD 2019 estimates from the Global Health Data Exchange available at the Institute for Health Metrics and Evaluation (IHME) website (http://ghdx.healthdata.org/gbd-results-tool) between 1990 and 2019. Under the ‘single’ tag, according to the research purpose, we can select indicators such as location, age, sex, year and measure. The extracted data were downloaded and saved in ‘CSV’ format. Data sorting and processing methods have been described in detail in previous studies [8–9]. All estimates were produced by the IHME [9], except for the average annual percentage change (AAPC) that was calculated by the authors.

The GBD employs standardised tools to model processed data and generate estimates of variables of interest by age, sex, location and year, such as the Cause of Death Ensemble model. A Bayesian meta-regression modeling tool (DisMod-MR 2.1) was used to ensure consistency between incidence, prevalence, remission, excess mortality and cause-specific mortality for most causes [9]. General methods for GBD 2019 and disease burden estimation have been detailed in previous studies [9, 10].

This study focuses on pancreatic cancer, defined according to the code C25 (ICD-10), for that was included countries with more than 30 cases in 2019, about 23 countries from 36 in LAC, was included comprising of Central America (Costa Rica, El Salvador, Guatemala, Honduras, Mexico, Nicaragua and Panama); the Caribbean (Cuba, Dominican Republic, Haiti, Jamaica, Puerto Rico and Trinidad and Tobago) and South America (Argentina, Bolivia, Brazil, Chile, Colombia, Ecuador, Paraguay, Peru, Uruguay and Venezuela). We included the results grouped into four regions according to the GDB regional grouping: Andean Latin America, Caribbean, Central Latin America and Tropical Latin America for both sexes (Supplementary Table 1).

We excluded countries with less than 30 cases in 2019, all of which were Caribbean countries, for a total of thirteen: Antigua and Barbuda, Bahamas, Barbados, Belize, Bermuda, Dominica, Guyana, Saint Kitts and Nevis, Saint Vincent and the Grenadines, Grenada, Saint Lucia, Suriname and United States Virgin Islands [9].

All estimates from GDB 2019 for countries in LAC between 1990 and 2019 were considered: the ASIR and the age-standardised mortality rate (ASMR), were obtained as adjusted rates and calculated per 100,000 inhabitants, and the DALYs, a composite indicator that expresses the total burden of diseases by combining in just one measure, the time individuals live with a certain disability, years lived with disability (YLD), prevalence estimates multiplied by disability weights for mutually exclusive sequelae of diseases and injuries, and the time lost due to premature mortality – years of Life Lost (YLL) – subtracting the age at death from the longest possible life expectancy for a person at that age. The proportion of pancreatic cancer age-standardised deaths attributable to behavioral (smoking) and metabolic (high fasting plasma glucose and high body-mass index) risk factors was also determined [9].

The AAPC in incidence and mortality adjusted rates in LAC, with respective 95% confidence intervals (95% CIs), were calculated, as well as the DALYs between 1990 and 2019, using the Joinpoint regression analysis and year as an independent variable. The AAPC measures trends over a period and describes the AAPC over several years using a single result number. For this analysis, the software used was the Joinpoint Regression Program version 4.9.0.0, March 2021, Statistical Research and Applications Branch, National Cancer Institute, Bethesda, USA [11].

The heat map was developed in Microsoft Excel. The maps were plotted in QGIS Development Team 2022 (version 3.24.2) Tisler from April 15, 2022, a software with a General Public License [12].

## Results

**Figure 1.**
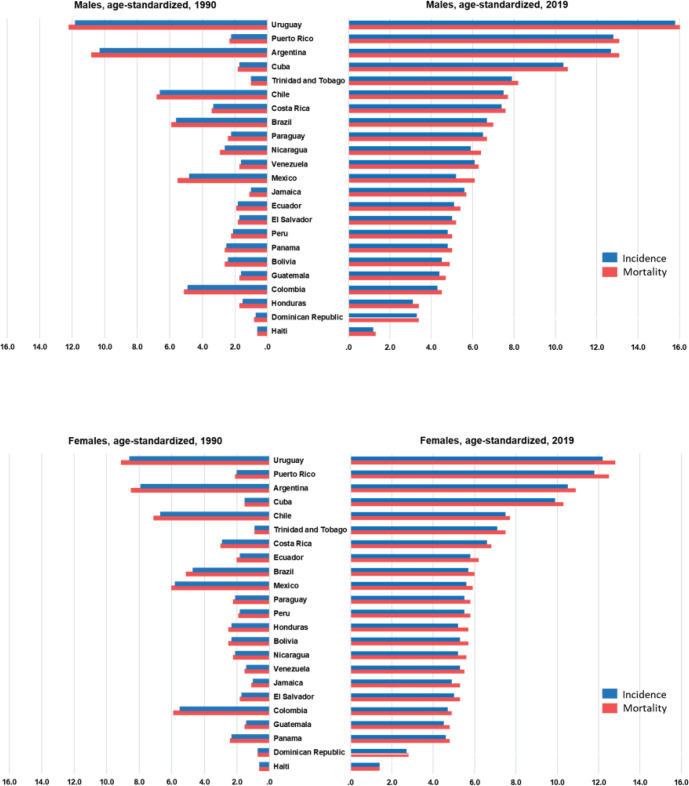
Age-standardised incidence and mortality distributed by sex in LAC countries in 1990 and 2019.

In LAC, most countries experienced increases in pancreatic cancer incidence, mortality trends and DALYs. In the 1990s, the incidence rates of pancreatic cancer ranged from 0.6/100,000 (Haiti) to 11.8/100,000 (Uruguay). Rates increased over time, and in 2019, the lowest rate remained in Haiti (1.2), whereas the highest registered rate was 15.8 in Uruguay, in men (Table 1, Figure 1). In women, the incidence ranged from 0.6/100,000 (Haiti) to 8.6 (Uruguay) in 1990. In 2019, it ranged from 1.4 (Haiti) to 12.2 (Uruguay) (Table 2, Figure 1).

**Figure 2.**
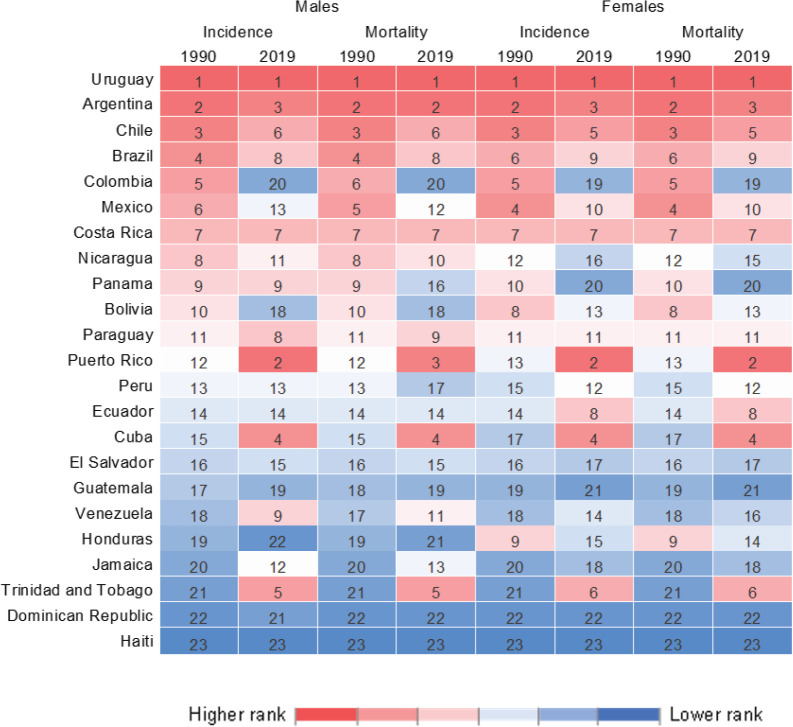
Heat map for incidence and mortality rank for pancreatic cancer in LAC countries in 1990 and 2019 by sex.

The top changes in rank occurred in Colombia (dropping from 5th in 1990 to 20th in 2019) and Puerto Rico (rising from 12th in 1990 to 2nd in 2019) (Figure 2).

**Figure 3.**
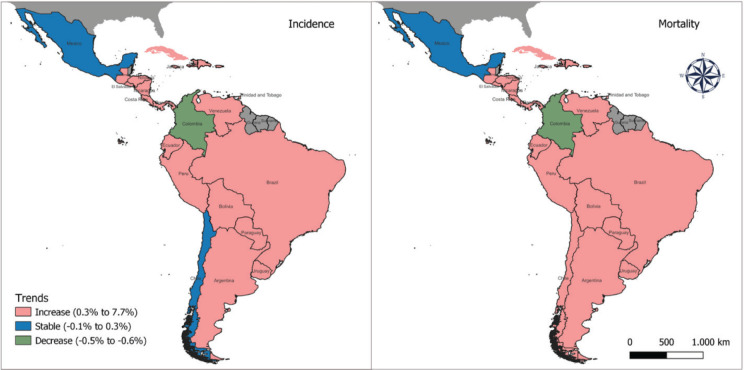
Incidence and mortality trends in LAC countries between 1990 and 2019 in both sexes.

The trends in pancreatic cancer incidence showed an increase in most countries in LAC, including 20 out of 23 countries. During the period studied, the AAPC ranged from 0.7% in Brazil (the lowest) to 7.7% in Trinidad and Tobago (the highest). Stable trends were observed in Mexico and Chile, whereas a decreasing trend was detected in Colombia (AAPC −0.5%, 95%IC −0.9; −0.1) for both sexes (Figure 3).

In LAC, pancreatic cancer mortality showed a higher incidence rate for both sexes: the highest mortality rate in 2019 was observed in Uruguay (16.3/100,000 among men, and 12.8/100,000 among women), followed by Puerto Rico (13.1, 12.5) and Argentina (13.1, 10.9). The lowest percentages were in the Dominican Republic (3.4, 2.8), Honduras (3.4, 5.7) and Haiti (1.3, 1.4; Tables 1 and 2; Figure 1). The mortality rank changed for Colombia from 5th in 1990 to 20th in 2019, whereas Puerto Rico shifted from 13th in 1990 to 2nd in 2019. The trends for pancreatic cancer mortality dropped in Colombia for both sexes (−0.5% 95%CI −0.9; −0.2 among men and −0.6% 95%CI −0.9; −0.4 among women) (Tables 1 and 2, Figure 3).

The age-standardised rates of DALYs for pancreatic cancer were higher in Uruguay, at 363.8/100,000 in 2019, with a 0.9% increase (95%CI: 0.8; 1.0), followed by Argentina (290.6, 2019), showing a yearly increase of 0.6% (95%CI 0.3;0.9). A contraction in DALYs was observed in Colombia for both sexes, from 120.2 in 1990 to 101.5 in 2019 among men, showing a decreasing trend by −0.8% (95%CI −1.2; −0.4); among women, from 127.7 in 1990 to 101.5 in 2019 illustrating a downward trend of −0.6% per year (95%CI −0.9; −0.4) (Tables 1 and 2;). The highest advance in DALYs was observed in Brazil (62,000 in 1990 to 176,000 in 2019) among men and from 52,000 to 163,000 among women, followed by Mexico, which registered DALYs at 27,000 to 80,000 among men. The YLL in Brazil, in 2019, was 174,583 among men, whereas, in Mexico, it was 79,816, and in women, the YLL was 162,085 in Brazil and 79,214 in Mexico (Supplementary Table 2).

**Figure 4.**
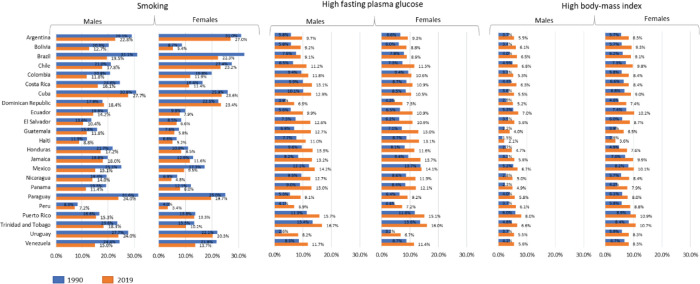
Fraction of pancreatic cancer age-standardised deaths attributable to smoking, high fasting plasma glucose and high body-mass index by LAC countries among males and females, 1990 and 2019.

The fraction of pancreatic cancer deaths attributable to smoking in LAC in 2019 was higher in Cuba (27.7%), followed by Paraguay and Uruguay with 24.0% among men, whereas among women, the smoking factor was higher in Argentina (27.0%), followed by Cuba (23.6%) and the Dominic Republic (23.4%). However, the proportion of smokers in LAC countries decreased between 1990 and 2019 for both sexes. For high fasting plasma glucose levels, the proportion of deaths was higher in Trinidad and Tobago (16.7% and 16.0%) followed by Puerto Rico (15.7%

for men and 15.1% for women). The prevalence of high body-mass index was 8.0% among men in Puerto Rico and 11.0% among women in Puerto Rico and Trinidad and Tobago (Figure 4).

## Discussion

In most LAC countries, an upward trend was observed for pancreatic cancer incidence and mortality. The proportion of pancreatic cancer deaths related to smoking reduced between 1990 and 2019 for both sexes, but increased for metabolic risk factors, such as high fasting plasma glucose and high body-mass index. The figures did not differ from the increasing trends observed worldwide [1–2, 4–5]. Uruguay had the highest incidence rates for both sexes in LAC, as described by Goodarzi *et al* [4]. Although the highest incidence rates of pancreatic cancer are reported in Asia, followed by Europe and North America, whereas the lowest is found in Oceania [13]. Incidence rates of pancreatic cancer are four to five times higher in areas with higher HDI, such as Europe, Northern America and Australia/New Zealand [1, 14], in comparison with LAC which has intermediate rates. The lowest incidence rates in LAC were observed in Haiti and the Dominic Republic, whereas in Brazil, the rates were similar to those in China (6.0/100,000) but not as high as the USA rates (10.1/100,000) [1, 15].

Incidence rates varied between LAC, which may be related to increased life expectancy, as well as changes in exposure to known risk factors. Aging is a non-modifiable risk factor, and pancreatic cancer is more frequent in people aged >50 years [3, 16, 17]. Changes in lifestyle in LAC have increased metabolic and behavioural risk factors, such as the prevalence of obesity, physical inactivity and consumption of saturated fats, high-calorie foods and type 2 diabetes mellitus [18–20]. Some studies have found an increased risk of pancreatic cancer among heavy drinkers of alcohol, but other studies have not found an association [21]. In addition to the aforementioned observations, changes in lifestyle and variations in incidence may also be related to the challenges of advances in pancreatic cancer diagnosis and treatment, which is still limited. Targeted group screenings (especially for those with a family history of this type of cancer) have been efficient for early-stage diagnosis, including blood markers for pancreatic cancer. But there is still no fully effective diagnostic biomarker [3, 22–24].

In addition to the lack of a specific biomarker, the main means of diagnosing this tumor are image screenings, such as nuclear magnetic resonance, which depend on the infrastructure of each country’s health system [25]. Richer countries with high/very high HDI tend to have more available tools and infrastructure. In addition, these regions have higher incidence and mortality rates for pancreatic cancer, as this disease is more common in developed countries. In 2019, Haiti, Mexico, Peru and Venezuela invested 5% of their Gross Domestic Product (GDP) in health, whereas Bolivia, Colombia, Costa Rica, Dominican Republic, Ecuador, El Salvador, Guatemala, Honduras, Jamaica, Nicaragua, Panama, Paraguay, Trinidad, and Tobago invested 6% to 8%. Argentina, Brazil, Chile and Uruguay invested above 9%, and Cuba 11%. In Japan, the United Kingdom, New Zealand and the USA, 10.7%, 10.2% and 9.7% of their GDP were invested, respectively [26]. In LAC, historically, these expenditures were mainly aimed at infectious diseases, relegating nontransmissible chronic diseases, such as cancer, to secondary plans [27].

In LAC mortality rates were higher than incidence rates, and the same inversion was described by a study of pancreatic cancer in Brazil and China [15] and other countries [2] with data from GBD 2019, which may be related to the methods employed by the GBD study for the correction/adjustments of the causes of mortality and the quality of existing incidence data, reflecting underreported cases, mostly through death certificates [28, 29]. Due to the high lethality of this disease, the ratio mortality: incidence (M:I) is close to or greater than 1 in all LAC cases, and most cases are registered from death certificates only [3]. The methods used by the GBD study to correct/adjust the causes of mortality and the quality of the existing incidence data may have contributed to an excess of deaths and/or evidenced the underestimation of the incidence [10]_._

Countries with higher rates of smoking often exhibit higher incidence and mortality rates for pancreatic cancer. Smoking is a well-established risk factor for pancreatic cancer, contributing significantly to the disease’s development [3–4, 30]. Studies have suggested an association between smoking prevalence and pancreatic cancer rates, indicating that countries with higher smoking rates tend to have higher rates of both incidence and mortality for this cancer. In 2019, the fraction of pancreatic cancer deaths attributable to smoking in LAC was higher in Cuba followed by Paraguay and Uruguay among men, while among women, this behavioural factor was higher in Argentina, Cuba and the Dominic Republic, but it was reduced between 1990 and 2019 for both sexes in LAC. While there was a rise for both sexes in high fasting plasma glucose and body-mass index for obesity. These factors may be associated with an increased incidence of pancreatic cancer and mortality. The variations in pancreatic cancer mortality in LAC can be attributed to differences in incidence, as well as data quality as changes in risk factors [30, 31].

DALYs estimates between 1990 and 2017 showed a 2.1-fold increase in pancreatic cancer rates [2]. In this study, we observed a three-fold increase in DALYs in Mexico among men and a 2.7-fold increase among women. In Brazil, the increase was 2.8-fold in DALYs among men and 3.1-fold among women. The above-described increases contributed considerably, mostly due to higher YLL, i.e., premature deaths [32]. Therefore, the prevalence of risk factors such as high Body mass index (BMI). and high glucose levels, may explain the observed increase in DALYs.

This study has some limitations, such as the validity and quality of the incidence and mortality estimates that depend on the degree of representativeness and quality of the information sources, which in general can be data from the Population-Based Cancer Registry and/or Information System on Mortality, as well as access to this data and estimation calculation methods [33–34]. Data on morphological classification were not available because some pancreatic cancers, such as neuroendocrine, have a better prognosis. Although they are rare, adenocarcinomas represent 90%–95% of all pancreatic cancer [35].

The increasing trends in the incidence, mortality and DALYs in LAC may be associated with an increase in metabolic risk factors such as high fasting plasma glucose and high body-mass index. More advances are needed in prevention, early diagnosis and treatment of pancreatic cancer, which are still limited. Therefore, behavioural and lifestyle changes for modifiable risk factors remain the best strategies for reducing disease burden. Since the burden of incidence, mortality and risk factors associated with pancreatic cancer will impact the LAC health system in the near future.

## Conflicts of interest

The authors declare no conflict of interest.

## Funding

This research received no specific grants.

## Author contributions

DMRS: Conceptualisation, Methodology, Data curation, Investigation, Formal analysis, Writing – original draft. MMO: Methodology, Validation, Writing – review and editing. GAP: Writing – review and editing. MPC: Conceptualisation, Methodology, Supervision, Writing – review and editing.

## Data sharing statement

The datasets were publicly available and referenced in the methods.

## Figures and Tables

**Table 1.** ASIR, ASMR and DALYs rates for pancreatic cancer for males, according to LAC countries in 1990 and 2019, and AAPC between 1990 and 2019.

	Incidence			Mortality			DALYs		
	1990	2019	AAPC (95%CI)	1990	2019	AAPC (95%CI)	1990	2019	AAPC (95%CI)
Country	^a^Rates (cases)	^a^Rates (cases)		^a^Rates (deaths)	^a^Rates (deaths)				
Central America									
Costa Rica	3.3 (27)	7.4 (172)	2.7* (1.3; 4.1)	3.4 (28)	7.6 (177)	2.7* (1.3; 4.0)	77.6	166.7	2.9* (2.2; 3.6)
El Salvador	1.7 (23)	5.0 (125)	3.8* (3.4; 4.2)	1.8 (23)	5.2 (130)	3.7* (3.4; 4.1)	41.9	122.1	3.8* (3.4; 4.1)
Guatemala	1.6 (28)	4.4 (216)	3.5* (2.9; 4.2)	1.7 (29)	4.7 (224)	3.6* (3.0; 4.2)	39.6	105.7	3.5* (3.0; 4.0)
Honduras	1.5 (15)	3.1 (86)	2.5* (1.8; 3.2)	1.7 (16)	3.4 (90)	2.4* (1.9; 3.0)	38.2	73.3	2.3* (2.2; 2.4)
Mexico	4.8 (93)	5.2 (356)	0.3 (−0.4; 0.9)	5.5 (93)	6.1 (358)	0.4* (0.1; 0.7)	117.7	125.5	0.3 (−0.5; 1.0)
Nicaragua	2.6 (17)	5.9 (108)	2.9* (2.4; 3.3)	2.9 (18)	6.4 (113)	2.8* (2.3;3.3)	60.6	129.4	2.6* (2.0; 3.2)
Panama	2.5 (18)	4.8 (95)	2.3* (1.9; 2.7)	2.6 (19)	5.0 (99)	2.3* (1.8; 2.8)	59.8	108.4	2.2* (1.7; 2.7)
The Caribbean									
Cuba	1.7 (93)	10.4 (585)	6.6* (5.9; 7.3)	1.8 (97)	10.6 (597)	6.5* (5.9; 7.2)	40.3	239.5	6.5* (5.9; 7.1)
Dominican Republic	0.7 (26)	3.3 (183)	5.5* (4.7; 6.4)	0.8 (27)	3.4 (187)	5.3* (4.8; 5.9)	20.0	87.6	5.2* (4.6; 5.8)
Haiti	0.6 (19)	1.2 (75)	2.7* (2.6; 2.9)	0.6 (19)	1.3 (76)	2.5* (2.4; 2.6)	17.5	34.5	2.4* (2.3; 2.5)
Jamaica	1.0 (12)	5.6 (78)	6.6* (4.1; 9.1)	1.1 (12)	5.7 (80)	6.4* (4.0; 8.9)	23.8	137.3	6.5* (4.1; 9.0)
Puerto Rico	2.2 (39)	12.8 (214)	6.3* (5.0; 7.7)	2.3 (41)	13.1 (220)	6.2* (5.0; 7.5)	52.8	273.3	5.9* (4.5; 7.3)
Trinidad and Tobago	1.0 (6)	7.9 (55)	7.7* (6.2; 9.1)	1.0 (6)	8.2 (57)	7.6* (6.4; 8.8)	25.1	194.2	7.5* (6.2; 8.8)
South America									
Argentina	10.3 (1,462)	12.7 (2,987)	0.8* (0.3; 1.3)	10.8 (1,506)	13.1 (3,057)	0.7* (0.4; 1.1)	247.4	290.6	0.6* (0.3; 0.9)
Bolivia	2.4 (35)	4.5 (186)	2.3* (2.0; 2.6)	2.6 (37)	4.9 (195)	2.2* (2.0; 2.4)	59.8	107.0	2.0* (1.8; 2.2)
Brazil	5.6 (2,308)	6.7 (7,089)	0.7* (0.6; 0.8)	5.9 (2,339)	7.0 (7,239)	0.6* (0.4; 0.7)	138.8	159.0	0.5* (0.3; 0.6)
Chile	6.6 (293)	7.5 (810)	0.5 (−0.3; 1.3)	6.8 (295)	7.7 (823)	0.5* (0.1; 1.0)	154.1	165.9	0.3 (−0.4; 1.0)
Colombia	4.9 (412)	4.3 (1,036)	−0.5* (−0.9; −0.1)	5.1 (418)	4.5 (1,065)	−0.5* (−0.9; −0.2)	120.2	101.5	−0.8* (−1.2; −0.4)
Ecuador	1.8 (46)	5.1 (358)	3.7* (3.3; 4.0)	1.9 (47)	5.4 (371)	3.7* (3.2; 4.2)	43.8	117.3	3.5* (3.1; 3.9)
Paraguay	2.2 (23)	6.5 (167)	3.8* (3.5; 4.2)	2.4 (23)	6.7 (171)	3.7* (3.3; 4.1)	53.8	151.2	3.7* (3.3; 4.0)
Peru	2.1 (120)	4.8 (738)	3.0* (2.3; 3.6)	2.2 (124)	5.0 (764)	2.9* (2.5; 3.4)	51.9	113.4	2.8* (2.2; 3.3)
Uruguay	11.8 (201)	15.8 (355)	1.0* (1.0; 1.1)	12.2 (205)	16.3 (367)	1.0* (0.9; 1.1)	280.0	363.8	0.9* (0.8; 1.0)
Venezuela	1.6 (72)	6.1 (820)	4.8* (3.9; 5.8)	1.7 (73)	6.3 (833)	4.7* (3.7; 5.7)	39.5	145.1	4.7* (3.7; 5.7)

^a^Adjusted rates per 100,000 inhabitants

*Statistically significant results

DALYs, Disability-Adjusted Life Years; AAPC, Average Annual Percent Change; CI, Confidence Interval

**Table 2.** ASIR, ASMR and DALYs rates for pancreatic cancer for females, according to LAC countries in 1990 and 2019, and AAPC between 1990 and 2019.

	Incidence			Mortality			DALYs		
	1990	2019	AAPC (95%CI)	1990	2019	AAPC (95%CI)	1990	2019	AAPC (95%CI)
Country	^a^Rates (cases)	^a^Rates (cases)		^a^Rates (deaths)	^a^Rates (deaths)				
Central America									
Costa Rica	2.9 (25)	6.6 (182)	3.0* (2.0; 4.1)	3.0 (26)	6.8 (190)	3.0* (2.2; 3.7)	63.9	138.4	2.7* (2.0; 3.5)
El Salvador	1.7 (26)	5.0 (173)	3.8* (3.3; 4.4)	1.8 (27)	5.3 (184)	3.8* (3.2; 4.4)	39.9	113.5	3.7* (3.1; 4.3)
Guatemala	1.4 (24)	4.5 (266)	4.0* (3.1; 4.9)	1.5 (25)	4.8 (278)	3.9* (2.9; 4.8)	33.1	104.0	4.0* (3.1; 4.8)
Honduras	2.3 (24)	5.2 (158)	3.0* (2.3; 3.8)	2.5 (25)	5.7 (167)	2.9* (2.3; 3.5)	55.5	117.5	2.6* (2.0; 3.3)
Mexico	5.8 (117)	5.6 (431)	−0.1 (−0.7; 0.5)	6.0 (120)	5.9 (440)	−0.1 (−0.3; 0.1)	133.2	125.9	−0.2 (−0.7; 0.3)
Nicaragua	2.1 (16)	5.2 (118)	3.1* (2.5; 3.8)	2.2 (17)	5.6 (125)	3.2* (2.6; 3.8)	49.0	111.2	2.8* (2.2; 3.4)
Panama	2.3 (17)	4.6 (98)	2.4* (1.5; 3.4)	2.4 (17)	4.8 (103)	2.2* (1.9; 2.5)	52.8	100.0	2.2* (1.6; 2.9)
The Caribbean									
Cuba	1.5 (79)	9.9 (566)	6.8* (6.2; 7.5)	1.5 (82)	10.3 (590)	7.0* (6.0; 7.9)	33.9	205.4	6.5* (5.7; 7.4)
Dominican Republic	0.7 (25)	2.7 (144)	4.9* (4.2; 5.5)	0.7 (26)	2.8 (152)	5.1* (4.7; 5.4)	17.1	63.8	4.6* (4.3; 5.0)
Haiti	0.6 (19)	1.4 (87)	2.9* (2.3; 3.5)	0.6 (19)	1.4 (89)	3.1* (2.8; 3.5)	19.9	36.6	2.9* (2.8; 3.1)
Jamaica	1.0 (12)	4.9 (70)	5.7* (5.1; 6.3)	1.1 (13)	5.3 (75)	5.6* (5.0; 6.2)	15.9	109.6	5.6* (5.1; 6.1)
Puerto Rico	2.0 (37)	11.8 (218)	6.5* (5.9; 7.1)	2.1 (39)	12.5 (231)	6.3* (4.9; 7.7)	43.7	219.2	5.7* (4.1; 7.2)
Trinidad and Tobago	0.9 (5)	7.1 (49)	7.6* (6.9; 8.4)	0.9 (6)	7.5 (52)	7.6* (7.0; 8.3)	21.5	154.6	7.2* (6.6; 7.9)
South America									
Argentina	7.9 (1,436)	10.5 (3,267)	1.0* (0.8; 1.3)	8.5 (1,529)	10.9 (3,456)	0.9* (0.7; 1.1)	176.5	224.1	0.8* (0.5; 1.2)
Bolivia	2.3 (39)	5.3 (236)	2.7* (2.5; 2.9)	2.5 (40)	5.7 (250)	2.9* (2.4; 3.4)	56.5	119.8	2.4* (2.2; 2.6)
Brazil	4.7 (2,123)	5.7 (7,341)	0.7* (0.6; 0.7)	5.1 (2,213)	6.0 (7,738)	0.6* (0.5; 0.7)	108.1	125.4	0.6* (0.5; 0.6)
Chile	6.7 (357)	7.5 (1,012)	0.4 (−0.0; 0.8)	7.1 (371)	7.7 (1,050)	0.3* (0.1; 0.6)	146.8	154.8	0.2 (−0.2; 0.5)
Colombia	5.5 (476)	4.7 (1,370)	−0.5* (−0.9; −0.1)	5.9 (493)	4.9 (1,430)	−0.6* (−0.9; −0.4)	127.7	104.7	−0.6* (−0.9; −0.4)
Ecuador	1.8 (47)	5.8 (441)	4.0* (3.5; 4.6)	2.0 (50)	6.2 (468)	4.1* (3.5; 4.6)	41.8	123.2	3.8* (3.2; 4.3)
Paraguay	2.1 (23)	5.5 (155)	3.3* (2.8; 3.9)	2.2 (25)	5.8 (164)	3.4* (3.0; 3.9)	47.8	119.6	3.2* (2.8; 3.6)
Peru	1.8 (108)	5.5 (909)	3.9* (3.3; 4.5)	1.9 (113)	5.8 (958)	3.9* (3.3; 4.5)	43.4	121.0	3.5* (2.4; 4.6)
Uruguay	8.6 (194)	12.2 (409)	1.3* (0.7; 1.8)	9.1 (206)	12.8 (444)	1.2* (0.8; 1.6)	191.1	257.6	1.0* (0.7; 1.3)
Venezuela	1.4 (69)	5.3 (805)	4.7* (3.9; 5.5)	1.5 (71)	5.5 (833)	4.6* (3.8; 5.4)	32.9	118.2	4.5* (3.7; 5.4)

^a^Adjusted rates per 100,000 inhabitants

*Statistically significant results

DALYs, Disability-Adjusted Life Years; AAPC, Average Annual Percent Change; CI, Confidence Interval

## Supplementary tables

**Supplementary Table 1.** Age-standardized incidence rates (ASIR), age-standardized mortality rates (ASMR) and DALYs rates for pancreatic cancer by sex, according to LAC regions in 1990 and 2019 and AAPC between 1990 to 2019.

	**Incidence**			**Mortality**			**DALYs**		
	1990	2019	AAPC (95%CI)	1990	2019	AAPC (95%CI)	1990	2019	AAPC (95%CI)
LAC regions	^a^Rates (cases)	^a^Rates (cases)		^a^Rates (deaths)	^a^Rates (deaths)				
Male									
Andean	2.1 (397)	4.1 (1,321)	2.4* (1.5; 3.4)	2.2 (421)	4.4 (1,405)	2.5* (1.7; 3.3)	58.6	105.7	2.1* (1.6; 2.6)
Caribbean	3.6 (631)	6.0 (1,403)	1.8* (1.5; 2.1)	3.9 (676)	6.3 (1,471)	1.7* (1.2; 2.2)	91.9	158.4	1.9* (1.5; 2.3)
Central	2.0 (1,651)	4.0 (4,904)	2.4* (1.9; 2.9)	2.1 (1,739)	4.2 (5,150)	2.4* (1.9; 2.8)	57.4	106.5	2.2* (1.9; 2.5)
Tropical	2.9 (2,202)	6.5 (7,149)	2.8* (2.6; 2.9)	3.0 (2,297)	6.9 (7,524)	2.9* (2.7; 3.0)	83.6	172.8	2.5* (2.4; 2.6)
									
Female									
Andean	2.5 (471)	5.3 (1.694)	2.7* (2.2; 3.2)	2.7 (510)	5.8 (1,835)	2.7* (2.2; 3.2)	64.4	128.5	2.4* (1.8; 3.0)
Caribbean	3.1 (560)	5.3 (1,259)	1.8* (1.5; 2.2)	3.4 (606)	5.7 (1,500)	1.8* (1.2; 2.4)	75.5	125.6	1.7* (1.3; 2.2)
Central	2.2 (1,854)	4.3 (5,560)	2.3* (2.2; 2.5)	2.4 (1,982)	4.6 (5,931)	2.3* (1.9; 2.7)	59.1	107.3	2.1* (1.8; 2.4)
Tropical	2.6 (1,982)	6.2 (7,124)	3.1* (3.0; 3.1)	2.7 (2,119)	6.7 (7,696)	3.2* (2.9; 3.5)	67.5	150.2	2.8* (2.5; 3.2)

^a^ Adjusted rates per 100,000 inhabitants.

*Statistically significant results

DALYs, Disability-Adjusted Life Years; AAPC, Average Annual Percent Change; CI, Confidence Interval

**Supplementary Table 2.** Numbers of YLD, YLL and DALYs by sex for 1990 and 2019 from pancreatic cancer in LAC countries.

	Males						Females					
	YLD		YLL		DALYs		YLD		YLL		DALYs	
Country	1990	2019	1990	2019	1990	2019	1990	2019	1990	2019	1990	2019
Argentina	295.2	586.7	36,332.7	68,493.0	36,627.9	69,079.7	284.4	623.5	31,513.6	65,774.9	31,798.0	66,398.4
Bolivia	7.5	39.7	944.3	4,638.4	951.9	4,678.1	8.2	49.0	988.2	5,557.2	996.4	5,606.2
Brazil	485.7	1,474.8	61,883.3	174,583.5	62,369.0	176,058.2	440.2	1,496.9	51,745.4	162,085.8	52,185.7	163,582.7
Chile	61.1	164.5	7152.6	18,083.8	7,213.7	18,248.3	71.0	198.2	8,005.7	20,141.6	8,076.7	20,339.7
Colombia	88.9	223.0	10,997.4	24,297.4	11,086.3	24,520.4	98.0	283.8	11,709.2	29,680.9	11,807.2	29,964.6
Costa Rica	5.9	34.9	667.6	3,974.9	673.5	4,009.8	5.3	36.6	574.8	3,802.2	580.1	3,838.8
Cuba	19.9	120.3	2,179.8	13,417,7	2,199.7	13,538.0	16.9	114.0	1,808.2	11,607.0	1.825,10	11,720.9
Dominican Republic	5.6	39.4	701.3	4,751.9	706.9	4,791.3	5.3	30.8	624.1	3,419.5	629.4	3,450.3
Ecuador	9.9	75.0	1,186.3	8,627.9	1,196.2	8,702.9	10.0	90.1	1,147.8	9,643.6	1,157.8	9,733.7
El Salvador	4.9	26.9	587.6	2,999.8	592.6	3,026.7	5.5	35.6	635.0	3,785.1	640.5	3,820.7
Guatemala	6.1	46.0	756.0	5,488.7	762.2	5,534.8	5.2	55.9	640.4	6,376.9	645.6	6,432.8
Haiti	4.1	16.1	535.1	2,061.8	539.3	2,077.9	4.0	18.6	514.2	2,318.5	518.2	2,337.1
Honduras	3.3	18.4	401.2	2,104.9	404.4	2,123.3	5.0	32.8	600.1	3,766.0	605.1	3,798.8
Jamaica	2.5	16.7	273.6	1,899.8	276.1	1,916.4	2.5	14.8	267.4	1,536.6	269.9	1,551.4
Mexico	220.8	676.4	27,237.6	79,816.5	27,458.4	80,492.9	245.3	714.8	29,068.7	79,214.6	29,314.0	79,929.4
Nicaragua	3.7	22.2	443.9	2,577.6	447.6	2,599.8	3.5	24.1	406.1	2,671.3	409.6	2,695.3
Panama	4.0	19.8	460.2	2,178.1	464.2	2,197.9	3.5	20.2	394.5	2,098.5	398.0	2,118.7
Paraguay	4.9	34.7	587.7	4,141.3	592.6	4,176.0	5.0	31.9	555.3	3,430.5	560.3	3,462.4
Peru	25.8	158.4	3,139.9	17,504.8	3,165.7	17,663.2	23.1	183.3	2,707.3	19,834.2	2,730.4	20,017.5
Puerto Rico	8.4	43.7	917.4	4,530.3	925.8	4,574.0	7.9	43.0	804.3	4,008.7	812.3	4,051.7
Trinidad and Tobago	1.3	11.4	149.5	1,340.4	150.8	1,351.8	1.1	9.9	127.9	1,058.8	129.0	1,068.7
Uruguay	39.9	68.9	4,746.6	7,880.8	4,786.6	7,949.7	38.1	76.0	4,084.7	7,602.3	4,122.8	7,678.3
Venezuela (Bolivarian Republic of)	15.6	173.4	1,888.5	20,429.4	1,904.1	20,602.8	14.7	167.9	1,719.4	18,294.4	1,734.1	18,462.2

DALYs, disability-adjusted life years; YLD, years lived with disability; YLL, years of life lost
